# Safety and Immunogenicity of the mRNA-1273 Coronavirus Disease 2019 Vaccine in Solid Organ Transplant Recipients

**DOI:** 10.1093/infdis/jiae140

**Published:** 2024-03-21

**Authors:** Amparo L Figueroa, Jamil R Azzi, Bijan Eghtesad, Frances Priddy, Dina Stolman, Uma Siangphoe, Iliana Leony Lasso, Elizabeth de Windt, Bethany Girard, Honghong Zhou, Jacqueline M Miller, Rituparna Das

**Affiliations:** Moderna, Cambridge, Massachusetts, USA; Brigham and Women's Hospital, Harvard Medical School, Boston, Massachusetts, USA; Cleveland Clinic, Cleveland, Ohio, USA; Moderna, Cambridge, Massachusetts, USA; Moderna, Cambridge, Massachusetts, USA; Moderna, Cambridge, Massachusetts, USA; Moderna, Cambridge, Massachusetts, USA; Moderna, Cambridge, Massachusetts, USA; Moderna, Cambridge, Massachusetts, USA; Moderna, Cambridge, Massachusetts, USA; Moderna, Cambridge, Massachusetts, USA; Moderna, Cambridge, Massachusetts, USA

**Keywords:** COVID-19, immunocompromised, mRNA-1273, solid organ transplant recipients, vaccine

## Abstract

**Background:**

Solid organ transplant recipients (SOTRs) are at high risk for severe COVID-19.

**Methods:**

This open-label, phase 3b trial evaluated mRNA-1273 in 137 kidney and 77 liver SOTRs and 20 immunocompetent participants. In part A, SOTRs received three 100-µg doses of mRNA-1273; immunocompetent participants received 2 doses. In part B, an additional 100-µg dose was offered ≥4 months after the primary series. Here, we report interim trial results.

**Results:**

mRNA-1273 was well-tolerated in SOTRs. Four serious adverse events were considered vaccine related by the investigator in 3 SOTRs with preexisting comorbidities. No vaccine-related biopsy-proven organ rejection events or deaths were reported. mRNA-1273 elicited modest neutralizing antibody responses after dose 2 and improved responses after dose 3 in SOTRs. Post–dose 3 responses among liver SOTRs were comparable to post–dose 2 responses in immunocompetent participants. Post-additional dose responses were increased in SOTRs, regardless of primary series vaccination. In liver SOTRs, post-additional dose responses were ∼3-fold higher versus post-dose 2 but lower than immunocompetent participant responses. Most kidney SOTRs received multiple immunosuppressants and had reduced antibody responses versus liver SOTRs.

**Conclusions:**

mRNA-1273 was well-tolerated, and dose 3 and the additional dose improved antibody responses among SOTRs.

**Clinical Trials Registration:**

NCT04860297.

## Graphical Abstract

**Graphical Abstract:**
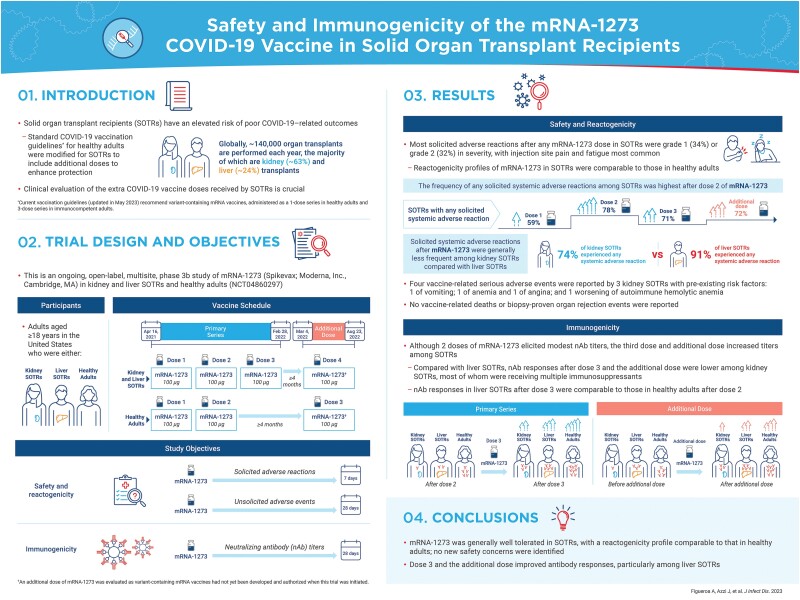

## Teaser

Messenger RNA–1273 (100 μg) administered as a 3-dose primary series and an additional dose was well tolerated among solid organ transplant recipients. Antibody responses were improved after dose 3 and the additional dose, supporting the recommended doses for this immunocompromised population.

Solid organ transplant recipients (SOTRs) are at high risk for infection-related disease and death [1, 2]. Increased susceptibility to infection arises from posttransplant immunosuppressive therapies (ISTs) that prevent organ rejection and enhance patient survival [1, 2]. However, IST use is associated with poor responses to vaccination in general [3], necessitating alternative vaccination strategies (eg, high-dose vaccination or additional doses) to protect against infections [4, 5].

During the coronavirus disease 2019 (COVID-19) pandemic, SOTRs experienced elevated rates of severe COVID-19–related outcomes compared with immunocompetent individuals [6–8]. High-risk populations, including SOTRs, were prioritized for vaccination after emergency use authorization of messenger RNA (mRNA) vaccines as a 2-dose schedule [9–11]. However, initial serosurveys and observational studies demonstrated seroconversion of only 54% among SOTRs after a 2-dose primary mRNA vaccine series compared with seroconversion rates of up to 100% among immunocompetent participants [12, 13]. Furthermore, SOTRs had a 10–485-fold higher risk of death after the primary series compared with the immunocompetent population.

To improve immune responses among SOTRs, amendments to vaccine schedules were warranted [14]. Updated guidelines for SOTRs now recommend a 3-dose primary series and additional dose/s with a variant-containing mRNA vaccine [15, 16]. Although immune responses are enhanced by a third COVID-19 vaccine dose among SOTRs who responded to a 2-dose primary series, a substantial proportion of SOTRs exhibit minimal responses after dose 3, including those who are older and have a higher degree of immunosuppression or lower estimated glomerular filtration rate [17]. Data describing immune responses after additional doses among SOTRs who previously received a 3-dose primary series are limited [18, 19]. Evaluation of additional protection that may be afforded by additional doses is crucial to inform future COVID-19 vaccine strategies for SOTRs and other comparable immunosuppressed populations. Herein, we report interim findings from the phase 3b trial assessing the safety and immunogenicity of mRNA-1273 (Spikevax; Moderna) administered as a 3-dose primary series and an additional dose in adult SOTRs aged ≥18 years.

## METHODS

### Trial Design and Participants

This open-label, phase 3b trial (NCT04860297; https://beta.clinicaltrials.gov/study/NCT04860297?term=NCT04860297%20&rank=1), conducted at 16 sites in the United States, included kidney and liver SOTRs and immunocompetent individuals. The trial was initiated in April 2021 after EUA of mRNA-1273 in US adults (aged ≥18 years) in December 2020. The study had 2 parts. Part A assessed 3-dose mRNA-1273 primary vaccination, and part B evaluated an additional mRNA-1273 dose in participants who had completed a primary COVID-19 vaccine series (3 doses of an mRNA vaccine [mRNA-1273 or BNT162b2]; 2 doses of a non-mRNA vaccine [Ad26.COV2.S]; or ≥1 dose of a non-mRNA vaccine combined with 1 dose of an mRNA vaccine). For part A, eligible participants were aged ≥18 years, had received a single kidney or liver transplant ≥90 days before enrollment, and were unvaccinated or previously vaccinated with 2 doses of mRNA-1273 after transplant. Immunocompetent healthy adults (aged ≥18 years) were eligible if they had not been vaccinated with any COVID-19 vaccine. For part B, participants were eligible if enrolled in part A and were ≥4 months from the last dose. SOTRs were also enrolled in part B if they met eligibility criteria for part A and had completed primary COVID-19 vaccination outside the study after transplant. Additional details and criteria are provided in the Supplement.

The study was conducted in accordance with the principles of the Declaration of Helsinki and applicable regulatory requirements. The study protocol, amendments, and informed consent forms were approved by an institutional review board. All participants provided written informed consent before any study procedure. Data presented here are based on the planned interim analysis (cutoff date 1 September 2022).

### Trial Vaccine

mRNA-1273 is a lipid nanoparticle–containing mRNA that encodes the spike glycoprotein of severe acute respiratory syndrome coronavirus 2 (SARS-CoV-2). A 100-µg mRNA-1273 dose (0.5 mL), the dose level authorized at the time of the study, was administered intramuscularly on days 1, 29, and 85 (part A) and on day 1 (part B; Supplementary Figure 1). An additional 100-µg dose was selected for 3 reasons: (1) the potential for reduced antibody titers compared with immunocompetent individuals due to chronic immunosuppression in SOTRs; (2) potential immune escape associated with emergent SARS-CoV-2 variants of concern; and (3) data demonstrating the effectiveness of a 50-µg dose in immunocompromised populations were not yet available at study initiation. In part A, unvaccinated SOTRs and immunocompetent participants received 2 doses of mRNA-1273 on days 1 and 29; SOTRs received dose 3 on day 85 (56 days after dose 2). In addition, SOTRs vaccinated with 2 doses of mRNA-1273 before the study received dose 3 on day 1. In part B, immunocompetent participants vaccinated with 2 doses of mRNA-1273 and SOTRs who completed primary mRNA or non-mRNA vaccination received an additional 100-µg dose on day 1.

### Study Objectives and End Points

Primary objectives included evaluation of the safety, reactogenicity, and immunogenicity of mRNA-1273 (100 µg) administered as a 3-dose primary series (part A) and an additional 100-µg dose (part B). Secondary objectives included evaluation of immune response persistence after dose 3. COVID-19 and severe COVID-19 incidence ≥14 days after vaccination were investigated as secondary (part A) and exploratory (part B) objectives.

### Assessments

Safety end points included solicited local and systemic adverse reactions (ARs) ≤7 days after vaccination; unsolicited adverse events (AEs) ≤28 days after vaccination; and medically attended AEs, serious AEs (SAEs), AEs of special interest, AEs leading to discontinuation, and biopsy-proven organ rejection throughout the study period. Suspected or confirmed events of organ rejection were reviewed by an adjudication committee; adjudicated biopsy-proven organ transplant rejection was reported as an SAE. Suspected cases of myocarditis and pericarditis were reviewed by an independent cardiac event adjudication committee, which operates under the rules of an approved charter.

Immunogenicity end points included neutralizing antibody (nAb) geometric mean concentrations (GMCs) and binding antibody (bAb) geometric means (GMs) through 6 months. Immunogenicity samples (blood) were collected before and 28 days and 6 months after each dose (Supplementary Figure 1). Additional details on assessments, including SARS-CoV-2 variant-specific bAb assessments, and symptomatic COVID-19 and severe COVID-19 case definitions, are in the Supplement.

### Statistical Analysis

There was no hypothesis testing in this study; the aim was to evaluate the safety and estimate immunogenicity of mRNA-1273. No statistical comparisons were planned. Immune responses were measured at prespecified time points (Supplementary Fig. 1). GMCs and GMs with corresponding 95% confidence intervals (CIs) are reported for nAbs and bAbs, respectively. Persistence of SARS-CoV-2–specific nAb and bAb was evaluated through 6 months after dose 3. Geometric mean fold rises (GMFRs) with corresponding 95% CIs are also reported. Seroresponse rates (SRRs), defined as the number of participants who achieved seroresponse (≥4-fold rise in antibody levels above baseline) were summarized and reported. Analysis populations and additional statistical methods are described in the Supplement.

## RESULTS

### Trial Population

**Figure 1.**
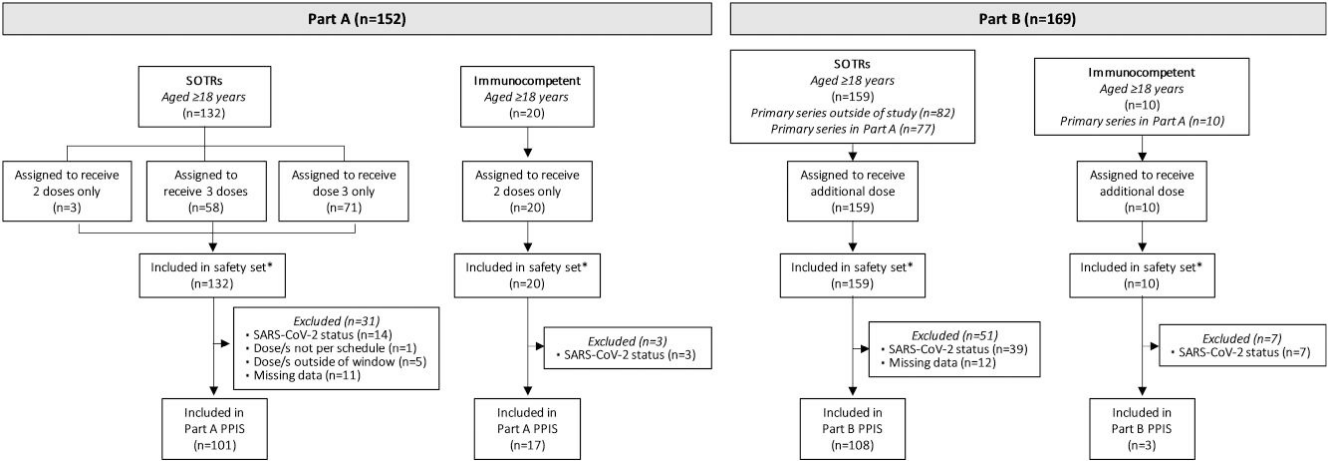
Participant enrollment and analysis populations. Enrollment of solid organ transplant recipients (SOTRs) and immunocompetent participants in part A took place between 16 April 2021 and 28 February 2022. SOTRs received a 3-dose primary series of mRNA-1273 or a third dose if previously vaccinated with 2 doses of mRNA-1273. Immunocompetent participants in part A received only 2 doses. SOTRs and immunocompetent participants from part A and SOTRs who previously completed primary vaccination outside of the study were enrolled in part B between 4 March and 23 August 2022 and received an additional dose. Participants with positive or missing severe acute respiratory syndrome coronavirus 2 (SARS-CoV-2) status at baseline (part A) or pre-additional dose (part B) were excluded. Other reasons for exclusion were doses not received per schedule (ie, doses not received or received out of window) and missing data (ie, pre- or postbaseline data [part A] or pre- or post-additional dose data [part B]). *Safety was assessed in the safety set combined for part A and part B, comprising a total of 214 SOTRs and 20 immunocompetent participants. Abbreviation: PPIS, per-protocol immunogenicity set.

Overall, 214 adult SOTRs (kidney, n = 137; liver, n = 77) and 20 adult immunocompetent participants were enrolled between 16 April 2021 and 28 February 2022 (part A) and between 4 March and 23 August 2022 (part B). In part A, 128 of 132 SOTRs (78 kidney and 50 liver SOTRs) received 3 doses (58 assigned to receive all 3 doses, and 71 receiving only dose 3 in the study) of mRNA-1273 and 20 immunocompetent participants received 2 doses (Figure 1). In part B, 159 SOTRs (98 kidney and 61 liver SOTRs) who completed primary series vaccination outside the study (n = 82) or in part A (n = 77) received an additional dose; 10 immunocompetent participants from part A received an additional dose. The median duration of safety follow-up in SOTRs (interquartile range [IQR]) was 292 (251–344) days after dose 3 and 129 (92–157) days after the additional dose.

Baseline demographics and clinical characteristics for the safety set (combined for both study parts) are shown in Table 1. Among SOTRs, the median age was 55.0 years; most were white (69.6%) and were kidney transplant recipients (64.0%). Concomitant combination therapy with mycophenolate, tacrolimus, and prednisone was more common in kidney (50.4%) than in liver (6.5%) SOTRs; tacrolimus monotherapy was more common in liver (44.2%) than in kidney (0%) SOTRs. A greater proportion of kidney than liver SOTRs received previous induction monotherapy with basiliximab (29.2% vs 11.7%) or thymoglobulin (28.5% vs 5.2%). The median age of immunocompetent participants was 41.5 years, and most were white (55.0%).

**Table 1.** Baseline Demographic and Clinical Characteristics (Safety Set)

Characteristic	SOTRs, No. (%)^a^			Immunocompetent Participants, No. (%)^a^ (n = 20)
	Total (n = 214)	Kidney (n = 137)	Liver (n = 77)	
Age at screening, mean (SD), y	52.5 (14.1)	52.0 (14.2)	53.2 (14.1)	44.0 (14.1)
Age distribution				
≥18 to <65 y	166 (77.6)	108 (78.8)	58 (75.3)	18 (90.0)
≥65 y	48 (22.4)	29 (21.2)	19 (24.7)	2 (10.0)
Male sex	114 (53.3)	75 (54.7)	39 (50.6)	10 (50.0)
Race and ethnicity^b^				
White	137 (64.0)	83 (60.6)	54 (70.1)	11 (55.0)
Communities of color	77 (36.0)	54 (39.4)	23 (29.9)	9 (45.0)
SARS-CoV-2–positive status				
Before vaccination^c^	9 (4.2)	6 (4.4)	3 (3.9)	3 (15.0)
Before additional dose^d^	36 (16.8)	20 (14.6)	16 (20.8)	6 (30.0)
Previous induction therapy				
Basiliximab	49 (22.9)	40 (29.2)	9 (11.7)	–
Thymoglobulin	43 (20.1)	39 (28.5)	4 (5.2)	…
Other^e^	105 (49.1)	53 (38.7)	52 (67.5)	–
Prior IST types^f^				
Calcineurin inhibitors	192 (89.7)	120 (87.6)	72 (93.5)	…
mTOR inhibitors	10 (4.7)	6 (4.4)	4 (5.2)	…
Antiproliferative agents	158 (73.8)	122 (89.1)	36 (46.8)	…
Steroids	122 (57.0)	108 (78.8)	14 (18.2)	…
Other	10 (4.7)	8 (5.8)	2 (2.6)	…
Concomitant ISTs^f^				
Antiproliferative	159 (74.3)	123 (89.9)	36 (46.8)	…
Calcineurin inhibitors	195 (91.1)	121 (88.3)	74 (96.1)	…
mTOR inhibitors	10 (4.7)	6 (4.4)	4 (5.2)	…
Steroids	126 (58.9)	109 (79.6)	17 (22.1)	…
Other	11 (5.1)	9 (6.6)	2 (2.6)	…
Concomitant combination ISTs^g^				
Mycophenolate, tacrolimus	39 (18.2)	20 (14.6)	19 (24.7)	…
Mycophenolate, tacrolimus, prednisone	74 (34.6)	69 (50.4)	5 (6.5)	…
Tacrolimus	34 (15.9)	0	34 (44.2)	…
Other	67 (31.3)	48 (35.0)	19 (24.7)	…
≥2 y Since transplantation	168 (78.5)	107 (78.1)	61 (79.2)	…
Vaccination status				
Dose 1	61 (28.5)	35 (25.5)	26 (33.8)	20 (100)
Dose 2	61 (28.5)	35 (25.5)	26 (33.8)	20 (100)
Dose 3	128 (59.8)	78 (56.9)	50 (64.9)	0
Additional dose	159 (74.3)	98 (71.5)	61 (79.2)	10 (50)
Type of primary series outside of the study^h^				
3-Dose mRNA-1273	36 (16.8)	21 (15.3)	15 (19.5)	…
3-Dose BNT162b2	41 (19.2)	32 (23.4)	9 (11.7)	…
3-Dose mRNA-1273-BNT162b2	3 (1.4)	1 (0.7)	2 (2.6)	…
2-Dose Ad26.COV2.S-mRNA-1273	2 (0.9)	2 (1.5)	0	…

Abbreviations: IST, immunosuppressive therapy; mRNA, messenger RNA; mTOR, mechanistic target of rapamycin; SARS-CoV-2, severe acute respiratory syndrome coronavirus 2; SD, standard deviation; SOTRs, solid organ transplant recipients.

^a^Data represent no. (%) of SOTRs or immunocompetent participants unless otherwise specified.

^b^White was defined as white and non-Hispanic; communities of color include all other participants whose race or ethnicity was not unknown, unreported, or missing.

^c^SARS-CoV-2–positive status before vaccination was assigned if a positive reverse-transcription polymerase chain reaction (RT-PCR) test or Elecsys result was obtained on day 1 before vaccination.

^d^SARS-CoV-2–positive status before an additional dose was assigned if a positive RT-PCR or Elecsys result was obtained on day 1, part B, before receipt of an additional dose.

^e^The “Other” category for previous induction therapy includes alemtuzumab, daclizumab, methylprednisolone, prednisone, combination therapy with basiliximab, and thymoglobulin.

^f^IST types included (1) calcineurin inhibitors: tacrolimus and cyclosporine; (2) mTOR inhibitors: everolimus and sirolimus; (3) antiproliferative agents: azathioprine and mycophenolate; (4) steroids: budesonide, methylprednisolone, and prednisone; and (5) other: adalimumab, belatacept, hydroxychloroquine, infliximab, and capecitabine.

^g^Concomitant combinations included tacrolimus, azathioprine, and prednisone; mycophenolate and prednisone; tacrolimus and azathioprine; and other rare combinations.

^h^Results are provided only for SOTRs who completed a primary series with mRNA (mRNA-1273 or BNT162b2) or non–mRNA (Ad26.COVS.2) combined with mRNA coronavirus disease 2019 vaccines outside of the study.

### Safety

**Figure 2.**
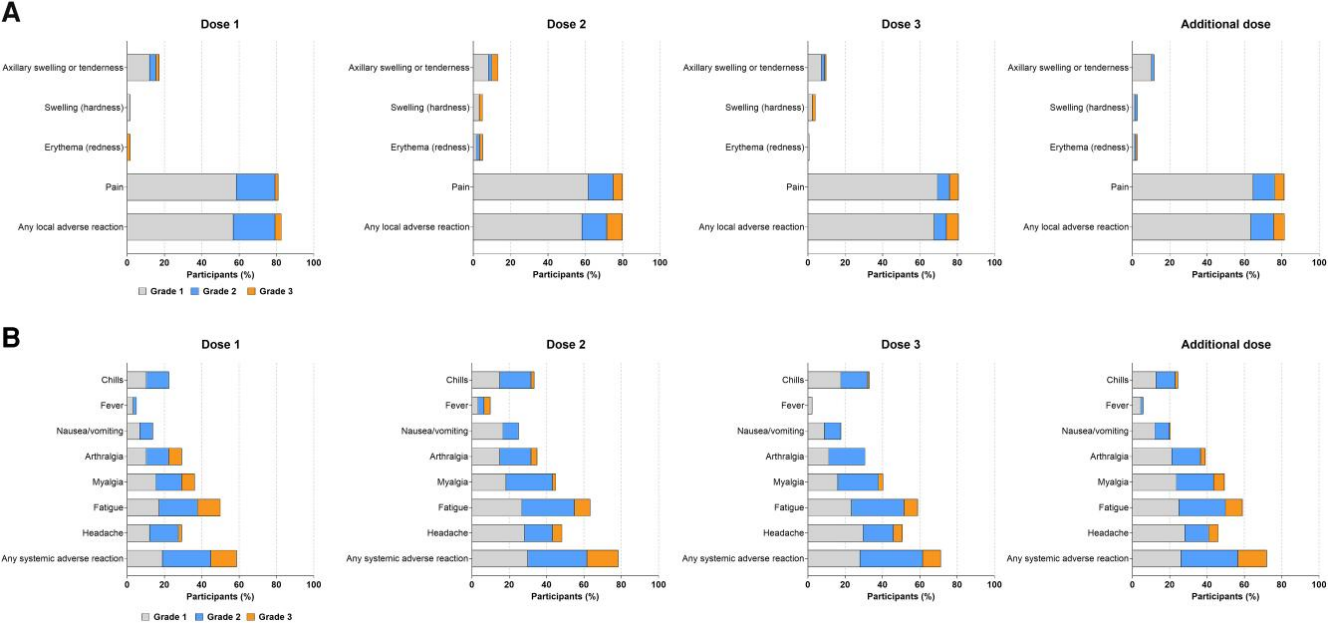
Local (*A*) and systemic (*B*) adverse reactions (ARs) by severity and dose among all solid organ transplant recipients (SOTRs; solicited safety sets). The percentage of SOTRs who reported solicited local and systemic ARs within 7 days of receiving mRNA-1273 in parts A and B are shown, stratified by grade and dose. Data are representative of the solicited safety sets, comprising all participants who reported a solicited AR after receipt of dose 1, dose 2, dose 3, or the additional dose during the study. The numbers of participants who reported data for any solicited AR were as follows: after dose 1, n = 58; after dose 2, n = 60; after dose 3, n = 125; after the additional dose, n = 157.

The safety set comprised 214 SOTRs (137 kidney and 77 liver SOTRs) and 20 immunocompetent participants from parts A and B combined. Solicited ARs within 7 days of any dose were reported for 93.0% of SOTRs; 84.9% and 80.3% reported local and systemic ARs, respectively. Injection site pain and fatigue were most commonly reported solicited local and systemic ARs, respectively (Figure 2). Most solicited ARs were grade 1 (34.3%) or 2 (31.5%) in severity, with a median (range) onset of 1 (1–3) day and duration of 4 (1–37) days. Solicited systemic ARs were more frequent after dose 2 than after dose 1, dose 3, or the additional dose. Kidney SOTRs reported fewer solicited ARs than liver SOTRs (90.4% vs 97.4%) across all doses, particularly for systemic ARs (74.3% vs 90.9%).

Unsolicited AEs were reported for 42.1% of SOTRs within 28 days of any dose; 21.5% were considered vaccine related (Supplementary Table 1). Most vaccine-related unsolicited AEs were also commonly reported as solicited ARs (eg, fatigue, headache, myalgia, and arthralgia). No vaccine-related safety concerns were identified. Four SAEs reported in 3 SOTRs were considered vaccine related by the investigator: 1 event of vomiting (day 5 after dose 2) in a kidney SOTR aged >50 years with a chronic pain disorder; 1 event of anemia and 1 event of angina (days 10 and 11 after additional doses, respectively) in a kidney SOTR aged >50 years with preexisting anemia and a history of hypertension; and 1 event of worsening of preexisting autoimmune hemolytic anemia (>100 days after dose 2) in a kidney SOTR aged >30 years with concurrent COVID-19 infection. Two SAEs with fatal outcomes occurred in participants with comorbid conditions: worsening congestive heart failure (>250 days after dose 3) in a kidney SOTR aged >65 years with a history of hypertension, cerebral infarction, and preexisting congestive heart failure; and cardiac arrest (>75 days after additional dose) in a kidney SOTR aged >70 years with hypertension and hyperlipidemia. Neither of the deaths were considered vaccine related by the investigator. Four cases of clinically suspected liver rejection were reported, none of which were related to mRNA-1273 per the investigator; 2 of these events were adjudicated as biopsy proven.

### Immunogenicity

The per-protocol immunogenicity analysis sets for part A and part B comprised 101 SOTRs (63 kidney and 38 liver SOTRs) and 17 immunocompetent participants for part A and 108 SOTRs (67 kidney and 41 liver SOTRs) and 3 immunocompetent participants for part B.

#### nAb Responses

**Figure 3.**
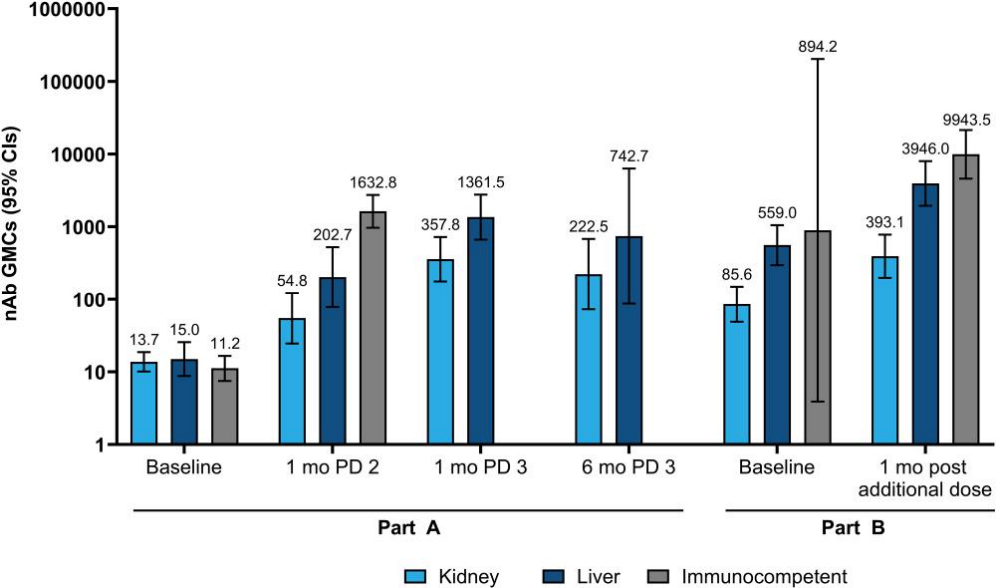
Neutralizing antibodies (nAbs) against ancestral severe acute respiratory syndrome coronavirus 2 (SARS-CoV-2) at baseline and after mRNA-1273 dose 2 and 3 (part A per-protocol immunogenicity set [PPIS]) and the additional dose (part B PPIS). nAbs were evaluated by means of pseudovirus neutralization assay at baseline (day 1 in part A; before additional dose in part B), 28 days (1 month) after dose 2 (PD 2) in part A, and 28 days (1 month) after the additional dose in part B in immunocompetent participants and solid organ transplant recipients (SOTRs). nAbs were also evaluated in part A 28 days (1 month) and 6 months after dose 3 (PD 3) in SOTRs; samples were not collected from immunocompetent participants at these time points because that they did not receive dose 3. Data are representative of the part A PPIS and the part B PPIS, comprising participants who tested negative for SARS-CoV-2 at the respective baseline, received vaccine doses according to schedule, and had no major protocol deviations. The numbers of participants with nonmissing data at the corresponding time points were as follows for part A: baseline, 40 SOTRs and 17 immunocompetent participants; 1 month after dose 2, 38 SOTRs and 17 immunocompetent participants; 1 month after dose 3, 81 SOTRs; and 6 months after dose 3, 31 SOTRs. The corresponding numbers for part B were as follows: baseline (pre-additional dose), 96 SOTRs and 3 immunocompetent participants; and 1 month after additional dose, 92 SOTRs and 3 immunocompetent participants. Data labels above each bar represent actual geometric mean concentrations (GMCs), and error bars represent 95% confidence intervals (CIs). Antibody values reported as below the lower limit of quantification (LLOQ) were replaced by 0.5 × LLOQ, and values above the upper limit of quantification (ULOQ) were replaced by the ULOQ.

In part A, nAb GMCs were not notably different between SOTRs and immunocompetent participants at baseline. A modest increase in nAb titers occurred 28 days after dose 2 relative to baseline in SOTRs (GMC, 88.7 [95% CI, 47.5–165.6]; GMFR, 6.1 [3.2–11.7]; Figure 3), which was lower than the increase in immunocompetent participants (GMC, 1632.8 [970.3–2747.8]; GMFR, 146.3 [86.2–248.3]).

Overall, nAb GMCs were improved among SOTRs after dose 3 (549.5; 95% CI, 320.4–942.3) and persisted above post–dose 2 levels at 6 months (Figure 3 and Supplementary Fig. 2). In sensitivity analyses that excluded SOTRs with postbaseline SARS-CoV-2 infection, nAb responses were comparable with those in the PPIS. Post–dose 3 nAb responses in kidney SOTRs were substantially improved relative to the 2-dose priming schedule; the fold rises in post–dose 3 nAb responses relative to post–dose 2 responses were comparable between kidney (6.5-fold) and liver (6.7-fold) SOTRs. However, GMCs were numerically higher in liver (1361.5 [95% CI, 668.4–2773.4]) than in kidney (357.8 [176.9–723.9]) SOTRs; GMCs in liver SOTRs were comparable to post–dose 2 levels in immunocompetent participants. In SOTRs, relative to post–dose 2 findings (liver, 10 of 14 [71.4%]; kidney, 11 of 24 [45.8%]), post–dose 3 SRRs were increased (liver, 11 of 12 [91.7%]; kidney, 16 of 23 [69.6%]).

In part B, pre–additional dose nAb GMCs were 85.6 (95% CI, 49.1–149.2) in kidney SOTRs, 559.0 (297.2–1051.4) in liver SOTRs, and 894.2 (3.9–203 262.7) in immunocompetent participants. GMCs were improved after the additional dose in kidney (393.1 [95% CI, 198.4–778.9]) and liver (3946.0 [1944.5–8007.8]) SOTRs (Figure 3 and Supplementary Figure 3). Post–additional dose nAb levels (GMFRs relative to the pre-additional dose) were substantially improved in kidney (4.2 [95% CI, 2.8–6.2]) and liver (7.0 [4.3–11.4]) SOTRs. However, GMCs were numerically higher in liver (3946.0 [95% CI, 1944.5–8007.8]) than in kidney (393.1 [198.4–778.9]) SOTRs. Post–additional dose nAb responses in liver SOTRs were about 3-fold higher than post–dose 3 levels but were still lower than in immunocompetent participants. Post–additional dose SRRs (relative to before) were similar for liver SOTRs (19 of 31 [61.3%]) and immunocompetent participants (2 of 3 [66.7%]) and higher than in kidney SOTRs (30 of 61 [49.2%]).

**Figure 4.**
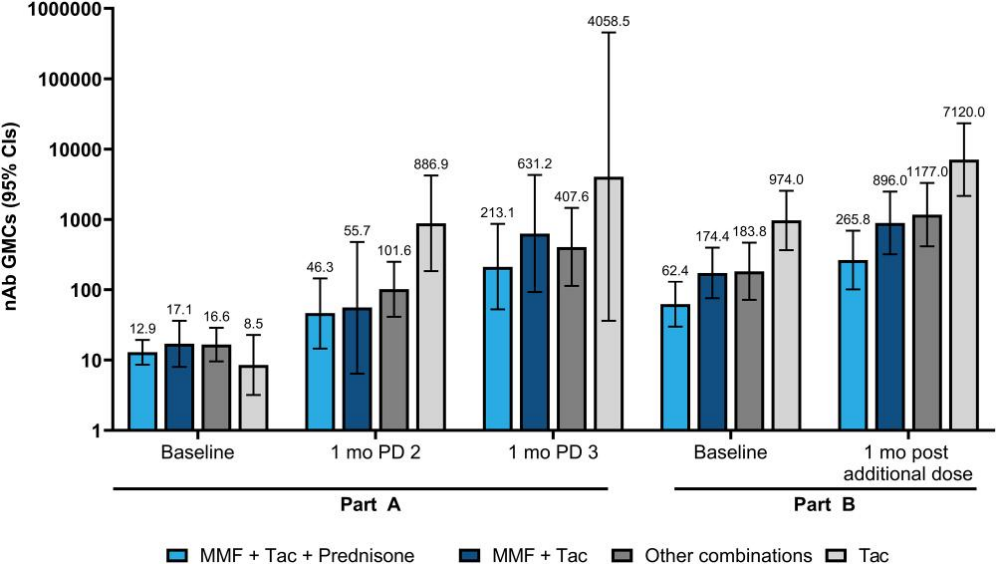
Neutralizing antibody (nAb) responses by immunosuppressive therapy (IST) regimen and dose among solid organ transplant recipients (SOTRs) (per-protocol immunogenicity set [PPIS]). nAbs were evaluated by means of pseudovirus neutralization assay at baseline (day 1 in part A; before additional dose in part B), and 28 days (1 month) after dose 2 (PD 2), 28 days (1 month) after dose 3 (PD 3), and 28 days (1 month) after the additional dose in SOTRs. Data are representative of the part A PPIS and part B PPIS, comprising participants who tested negative for severe acute respiratory syndrome coronavirus 2 (SARS-CoV-2) at the respective baseline, received vaccine doses according to schedule, and had no major protocol deviations. The IST regimens at each time point were as follows for part A: baseline, mycophenolate mofetil (MMF) + tacrolimus (Tac) + prednisone (n = 15), MMF + Tac (n = 6), other combinations (n = 15), and Tac (n = 4); 1 month after dose 2, MMF + Tac + prednisone (n = 13), MMF + Tac (n = 6), other combinations (n = 15), and Tac (n = 4); and 1 month after dose 3, MMF + Tac + prednisone (n = 12), MMF + Tac (n = 6), other combinations (n = 14), and Tac (n = 3). The regimens for part B were as follows: baseline, MMF + Tac + prednisone (n = 33), MMF + Tac (n = 20), other combinations (n = 29), and Tac (n = 14); and 1 month after additional dose, MMF + Tac + prednisone (n = 32), MMF + Tac (n = 19), other combinations (n = 28), and Tac (n = 13). Data labels above each bar represent actual geometric mean concentrations (GMCs), and error bars represent 95% confidence intervals (CIs). Antibody values reported as below the lower limit of quantification (LLOQ) were replaced by 0.5 × LLOQ, and values above the upper limit of quantification (ULOQ) were replaced by the ULOQ. Other combinations included combinations of Tac, azathioprine, and prednisone; mycophenolate and prednisone; Tac and azathioprine; other rare combinations; and other rare single ISTs.

In subgroup analyses, post–dose 3 and post–additional dose nAb levels were reduced relative to the respective baseline measurement in SOTRs receiving antimetabolite immunosuppressants compared with those not using this IST (Supplementary Table 2). Likewise, combination therapy (>1 IST) was associated with lower post–dose 3 and post–additional dose nAb responses compared with single IST regimens (Figure 4). In part B, subgroup analyses by primary series type (ie, 3 doses of mRNA-1273 or BNT162b2; 2 doses of Ad26.COV2.S; ≥1 non-mRNA dose combined with an mRNA vaccine dose) among SOTRs showed improved nAb responses after the additional dose versus pre–additional dose levels, irrespective of the primary series received (Supplementary Table 3).

#### Antibody Responses Against SARS-CoV-2 Variants of Concern

Similar trends were observed for bAb responses against variants of concern. Three doses improved bAb responses in SOTRs for the Alpha, Beta, and Gamma variants, as well as Delta (GM, 42 828.9 [95% CI, 23 029.2–79 651.8]; GMFR relative to baseline, 321.0 [124.4–828.0]) and Omicron (BA.1; GM, 12 024.7 [6581.6–21 969.5]; GMFR, 25.2 [10.6–60.0]). Comparatively, the additional dose modestly increased bAb responses compared with the pre–additional dose baseline in SOTRs for Delta (GM, 77 081.0 [95% CI, 44 726.7–132 839.5]; GMFR relative to pre–additional dose baseline, 6.7 [95% CI, 5.1–8.8]) and Omicron (GM, 2749.6 [1585.5–4768.5]; GMFR, 6.6 [5.0–8.8]). Generally, bAb GMs against variants were higher among liver than among kidney SOTRs after dose 3 and the additional dose; bAb levels were numerically lower against Omicron compared with the Alpha, Beta, Gamma, and Delta variants.

### COVID-19 and Severe COVID-19 Incidence Rates

COVID-19 occurred in 14.8% of SOTRs after dose 3 and 5.0% of SOTRs after the additional dose (incidence rates, 19.8 and 12.4 per 1000 person-months, respectively). COVID-19 occurred in 11.8% of immunocompetent participants after dose 2 (11.7 per 1000 person-months), with no cases reported after the additional dose. Severe COVID-19 occurred in 2.6% of SOTRs after dose 3 and 1.7% of SOTRs after the additional dose (3.2 and 4.0 per 1000 person-months, respectively); no cases were reported among immunocompetent participants. No COVID-19–related deaths were reported during the study.

## DISCUSSION

In this open-label, phase 3b trial, mRNA-1273 (100 µg)—administered as a 3-dose primary series and an additional dose—was well tolerated among SOTRs with no vaccine-related safety concerns. The reactogenicity profile of mRNA-1273 in SOTRs was consistent with that in immunocompetent participants from the phase 3 COVE trial [20, 21]. Antibody responses were improved after dose 3 and the additional dose, particularly among liver SOTRs, for whom responses were similar to those reported here and elsewhere for immunocompetent participants after dose 2 and the additional dose, respectively [20, 22]. These results underscore the importance of additional COVID-19 vaccine doses recommended for SOTRs.

Two mRNA-1273 doses elicited modest increases in nAb levels relative to baseline in SOTRs, congruent with previous studies [13, 23, 24]. The bAb responses followed similar trends and were consistent with previous clinical trial results [25, 26]. Post–dose 2 bAb GMs against variants, including Delta and Omicron, exhibited modest increases relative to baseline; however, these responses were subsequently improved by a third mRNA-1273 dose. Generally, post–dose 3 and post–additional dose bAb GMs against variants were higher among liver than kidney SOTRs. Furthermore, our data demonstrate that improved post–dose 3 responses persisted through 6 months in SOTRs; however, compared with liver SOTRs, nAb titers were lower among kidney SOTRs, most of whom were receiving combination immunosuppressants. Most liver SOTRs did not receive combination ISTs, partly explaining the more robust immune response improvement. More frequent combination IST use may also underlie the lower frequency of solicited systemic ARs reported for kidney versus liver SOTRs.

Post–dose 3 SRRs for liver and kidney SOTRs were consistent with previous reports [26–28]. In addition, our observations are congruent with previous studies demonstrating mRNA COVID-19 vaccine immunogenicity to be highly dependent on the type of concomitant therapy received by SOTRs. Although limited by relatively small sample sizes when stratified by IST use, numerically lower nAb responses were observed after mRNA-1273 among SOTRs receiving antimetabolites or triple combination regimens compared with other IST types. This reflects findings from other studies in SOTRs (including heart, lung, and pancreas transplant recipients) in which these ISTs were also associated with low response or nonresponse to mRNA vaccination [13, 24, 29, 30].

Currently, post–additional dose immune response data for SOTRs who previously received a 3-dose primary COVID-19 vaccine series are limited. Nevertheless, small studies suggest moderate immune response improvements among SOTRs with weak responses after an additional mRNA vaccine dose [18, 19]. Among 37 SOTRs (68% kidney SOTRs), anti–SARS-CoV-2 antibodies were detected in 13.5% (5 of 37) and 48.6% (18 of 37) before and 1 month after an additional BNT162b2 dose, respectively [18]. Among 6 seronegative SOTRs and 2 with low-positive titers after a 3-dose primary series, 63% (5 of 8) had high-positive titers after an additional dose of mRNA-1273, BNT162b2, or Ad26.CoV2.S; most SOTRs with high-positive post–dose 3 titers showed further improvements after the additional dose [19].

Given the urgency of enhancing protection against the Omicron variant at the time of study initiation, the additional mRNA-1273 dose was evaluated at a higher dose (100 µg) than the 50-µg dose of the previously authorized original mRNA-1273 vaccine and currently authorized variant-containing vaccine. Importantly, a 100-µg dose was selected based on authorization of mRNA-1273 at this dose level at study initiation; furthermore, effectiveness data for the 50-µg dose were not available for immunocompromised populations during the additional dose administration period in the study. The 50-µg dose was not evaluated herein; however, effectiveness data for a 50-µg dose among immunocompromised individuals are now available.

A recent, large, prospective study conducted during Omicron BA.5 and BQ.1 predominance evaluated the effectiveness of an additional dose (50 µg) of variant-containing vaccine, mRNA-1273.222 against COVID-19–related hospitalization after ≥2 original mRNA vaccine doses in immunocompromised individuals [31]. The relative vaccine effectiveness (VE) of mRNA-1273.222 versus an original mRNA vaccine was comparable between immunocompromised (64.7% [95% CI, 44.0%–77.7%]) and immunocompetent (71.3% [64.5%–76.7%]) individuals; the absolute VE was 71.8% (95% CI, 48.8%–84.5%) and 84.1% (80.1%–87.4%), respectively. In a VISION Network data study, the estimated VE against confirmed COVID-19–related hospitalization during Omicron BA.4/BA.5 and XBB-related predominance was notably lower 7–59 days after a variant-containing mRNA vaccine dose; the respective VEs were 28% (95% CI, 10–42) and 62% (57–67) among immunocompromised and immunocompetent individuals who were previously vaccinated with 1–5 doses of an mRNA vaccine [32]. However, additional VISION Network analyses that evaluated a third original mRNA-1273 or BNT162b2 dose during Delta predominance reported comparable VEs against hospitalization among immunocompromised (81% [95% CI, 76%–86%]) and immunocompetent (95% [94%–96%]) individuals [33].

The discrepancies in VE between studies may partly be explained by differences in sample size, period of follow-up, and methods to define COVID-19 hospitalization outcomes. The specific vaccine received may also play a role; studies of immunocompromised individuals have reported differences in VE against hospitalizations between mRNA-1273 and BNT162b2 [33, 34].

In summary, a 3-dose primary series and additional dose of mRNA-1273 had an acceptable safety profile and no risk of vaccine-related organ rejection in kidney and liver SOTRs; responses were observed, although SOTRs receiving combination ISTs (mostly kidney SOTRs) had reduced responses. The data presented here for mRNA-1273 are relevant to variant-containing COVID-19 vaccines as they were designed and synthesized using the same mRNA vaccine platform as the original vaccine. However, given the abovementioned limitations and variability between real-world effectiveness studies, continued assessment of protection against COVID-19–related hospitalizations in SOTRs following administration of variant-containing vaccines is needed.

Study strengths include the extended safety and immunogenicity follow-up period through 6 months after dose 3 and the use of quantitative immunoassays for nAb and bAb evaluation. The inclusion of participants who received heterologous primary vaccination outside of the study allowed findings to represent primary vaccination status more accurately in the real-world SOTR population. Limitations include the absence of a 50-µg additional dose and an unvaccinated SOTR population for comparison, the open-label study design, and the small sample of SOTRs that did not include other transplant types (eg, heart and lung transplant recipients) thereby limiting COVID-19 incidence rate assessments and subgroup analyses. In addition, most SOTRs had received their transplant ≥2 years earlier, limiting extrapolation of the findings to SOTRs who are within the posttransplant period associated with greatest vulnerability to infection (<1 year) [2, 35, 36]. Although only humoral immunogenicity was reported herein, samples were collected to evaluate cell-mediated responses to mRNA-1273 in SOTRs.

These results support the known benefits of COVID-19 vaccination for SOTRs and may also inform vaccine recommendations for comparable immunosuppressed populations. Our study contributes to the currently limited knowledge of COVID-19 vaccine performance in immunocompromised populations and demonstrates the importance of the extended primary and additional COVID-19 vaccination doses for SOTRs.

## Supplementary Data

Supplementary materials are available at *The Journal of Infectious Diseases* online (http://jid.oxfordjournals.org/). Supplementary materials consist of data provided by the author that are published to benefit the reader. The posted materials are not copyedited. The contents of all supplementary data are the sole responsibility of the authors. Questions or messages regarding errors should be addressed to the author.

## Supplementary Material

jiae140_Supplementary_Data

## Notes

***Acknowledgments.*** Medical writing and editorial assistance were provided by Jessica Nepomuceno, PhD, of MEDiSTRAVA in accordance with Good Publication Practice (GPP 2022) guidelines, funded by Moderna, Inc., and under the direction of the authors.

***Author contributions***. A. L. F., J. R. A., D. S., U. S., H. Z., J. M. M., and R. D. contributed to concept design. All authors contributed to the acquisition, analysis, or interpretation of the data and to writing and/or reviewing for intellectual contribution, and all authors approved the final draft.

***Data availability***. Access to patient-level data presented in the article and supporting clinical documents by qualified external researchers who provide methodologically sound scientific proposals may be available on reasonable request and subject to review. Such requests can be made to Moderna, Inc., 325 Binney Street, Cambridge, MA 02142. A materials transfer and/or data access agreement with the sponsor will be required for accessing shared data. All other relevant data are presented in the article.

***Financial support*.** This work was supported by Moderna, Inc.
