# Supplementary material for: Safety and Immunogenicity of the mRNA-1273 Coronavirus Disease 2019 Vaccine in Solid Organ Transplant Recipients
Source: J Infect Dis. 2024 Mar 21;230(3):e591–600. doi: 10.1093/infdis/jiae140 (PMC11420796; doi:10.1093/infdis/jiae140)

**Fig. S1. Overview of the study design**. In Part A, unvaccinated SOTRs and immunocompetent participants received 2 doses of mRNA-1273 on Days 1 and 29; unvaccinated SOTRs received dose 3 on Day 85 (56 days post-dose 2). Additionally, SOTRs in Part A vaccinated with 2 doses of mRNA-1273 prior to the study received dose 3 on Day 1. In Part B, immunocompetent participants vaccinated with 2 doses of mRNA-1273 and SOTRs who completed primary mRNA or non-mRNA vaccination received an additional dose on Day 1. SARS-CoV-2 infection was monitored and clinically assessed by the investigator by the collection of nasopharyngeal swabs at the pre-specified study visits. Safety laboratory test samples were obtained to help monitor for potential organ transplant rejection among SOTRs and included assessments related to kidney and liver functions. Safety was assessed in the safety set, which included participants who received mRNA-1273 in Part A or Part B. Safety outcomes were also evaluated in the solicited safety set, which comprised participants contributing any solicited adverse reactions after any mRNA-1273 dose. Immunogenicity was assessed in the respective Part A and Part B per-protocol immunogenicity sets (PPISs), which comprised participants in the safety set without SARS-CoV-2 infection pre-vaccination (Part A) and before the additional dose (Part B), received planned doses per schedule, and had no major protocol deviations. COVID-19 and severe COVID-19 incidences were assessed in the modified intent-to-treat sets, which comprised all participants in the safety set without SARS-CoV-2 infection pre-vaccination (Part A) and before the additional dose (Part B). D, day; SOTR, solid organ transplant recipient.


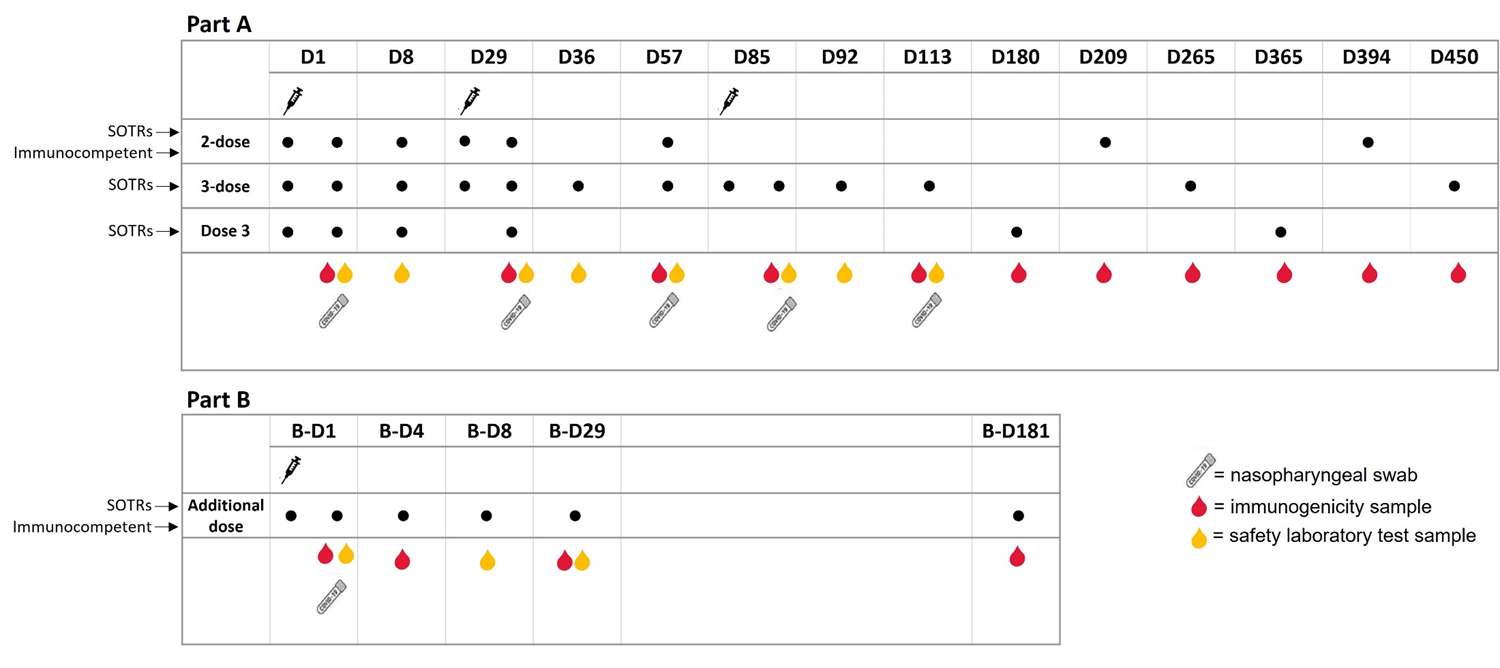

Supplement: jiae140_Supplementary_Data [file jiae140_supplementary_data.zip › Figueroa_Supplementary Figure 1_Revised.docx]
