# Supplementary material for: Safety and Immunogenicity of the mRNA-1273 Coronavirus Disease 2019 Vaccine in Solid Organ Transplant Recipients
Source: J Infect Dis. 2024 Mar 21;230(3):e591–600. doi: 10.1093/infdis/jiae140 (PMC11420796; doi:10.1093/infdis/jiae140)
Supplement: jiae140_Supplementary_Data [file jiae140_supplementary_data.zip › Figueroa_Supplementary_Figure_S2_JID.docx]

**Fig.** S2. Reverse cumulative distribution function plots of nAb concentrations (Part A PPIS). Reverse cumulative distribution function plots of pseudovirus nAb concentrations for the unvaccinated SOTRs who received up to 3 doses of mRNA-1273 and healthy participants who received 2 doses of mRNA-1273 in the study in the Part A PPIS are presented. The number of participants at each timepoint shown was as follows: Baseline: kidney, n=25; liver, n=15; 1 month post-dose 2 (Day 57): kidney, n=24; liver, n=14; 1 month post-dose 3 (Day 113): kidney, n=23; liver, n=12; 6 months post-dose 3 (Day 265): kidney, n=12; liver, n=6. Antibody values reported as below the LLOQ were replaced by 0.5 x LLOQ, and values greater than the ULOQ were replaced by the ULOQ if actual values were not available. LLOQ, lower limit of quantification nAb, neutralizing antibody; PPIS, per-protocol immunogenicity set; SOTR, solid organ transplant recipient; ULOQ, upper limit of quantification.

***
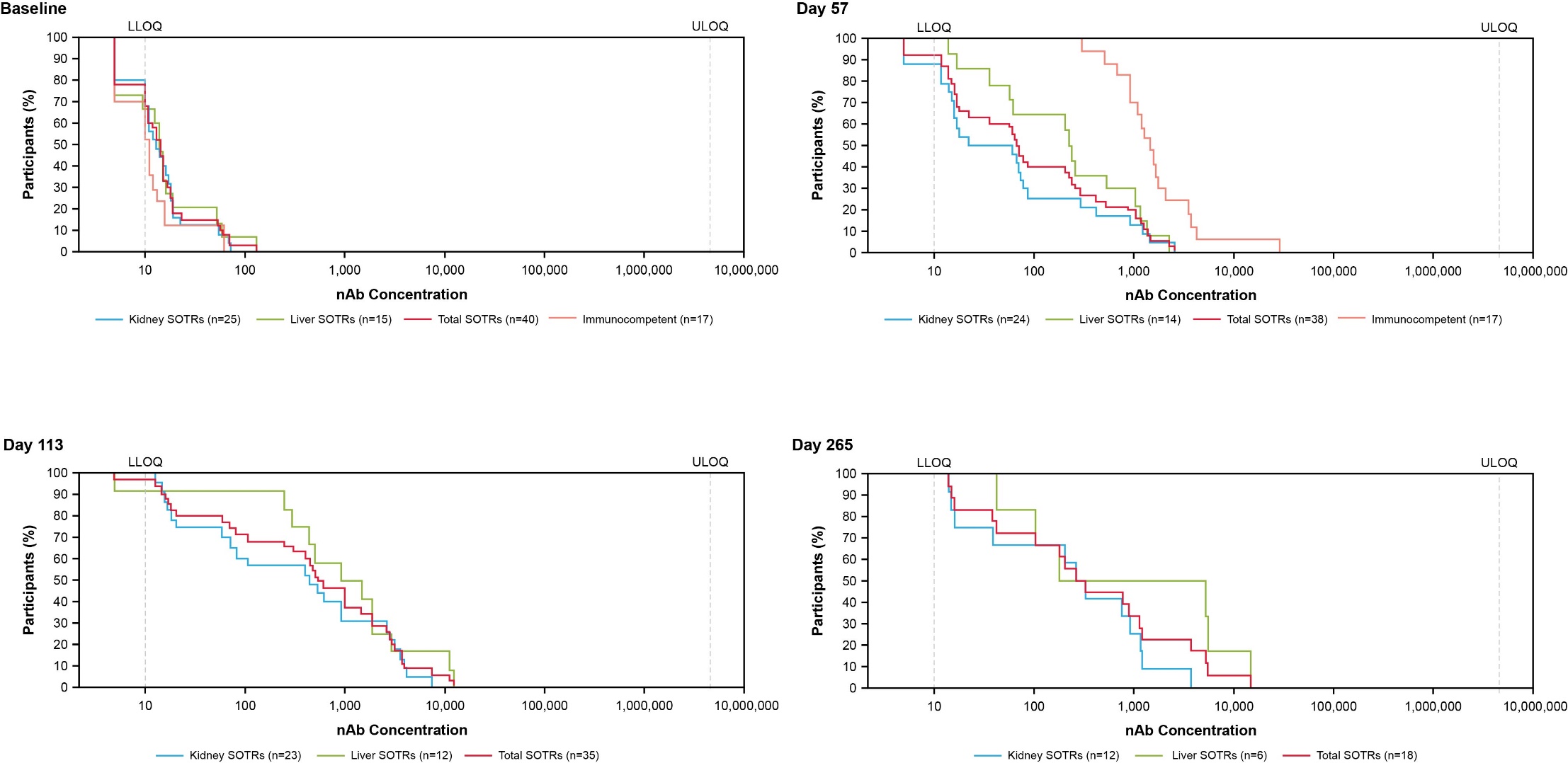
***
