# Supplementary material for: Safety and Immunogenicity of the mRNA-1273 Coronavirus Disease 2019 Vaccine in Solid Organ Transplant Recipients
Source: J Infect Dis. 2024 Mar 21;230(3):e591–600. doi: 10.1093/infdis/jiae140 (PMC11420796; doi:10.1093/infdis/jiae140)

**Fig. S3. Reverse cumulative distribution function plots of nAb concentrations (Part B PPIS).** Reverse cumulative distribution function plots of pseudovirus nAb concentrations for the SOTRs who received mRNA-1273 in the Part B PPIS are presented. The number of participants at each timepoint shown was as follows: Baseline: kidney, n=64; liver, n=32; 1 month post-additional dose: kidney, n=61; liver, n= 31. Antibody values reported as below the LLOQ were replaced by 0.5 × LLOQ, and values greater than the ULOQ were replaced by the ULOQ. LLOQ, lower limit of quantification; nAb, neutralizing antibody; PPIS,


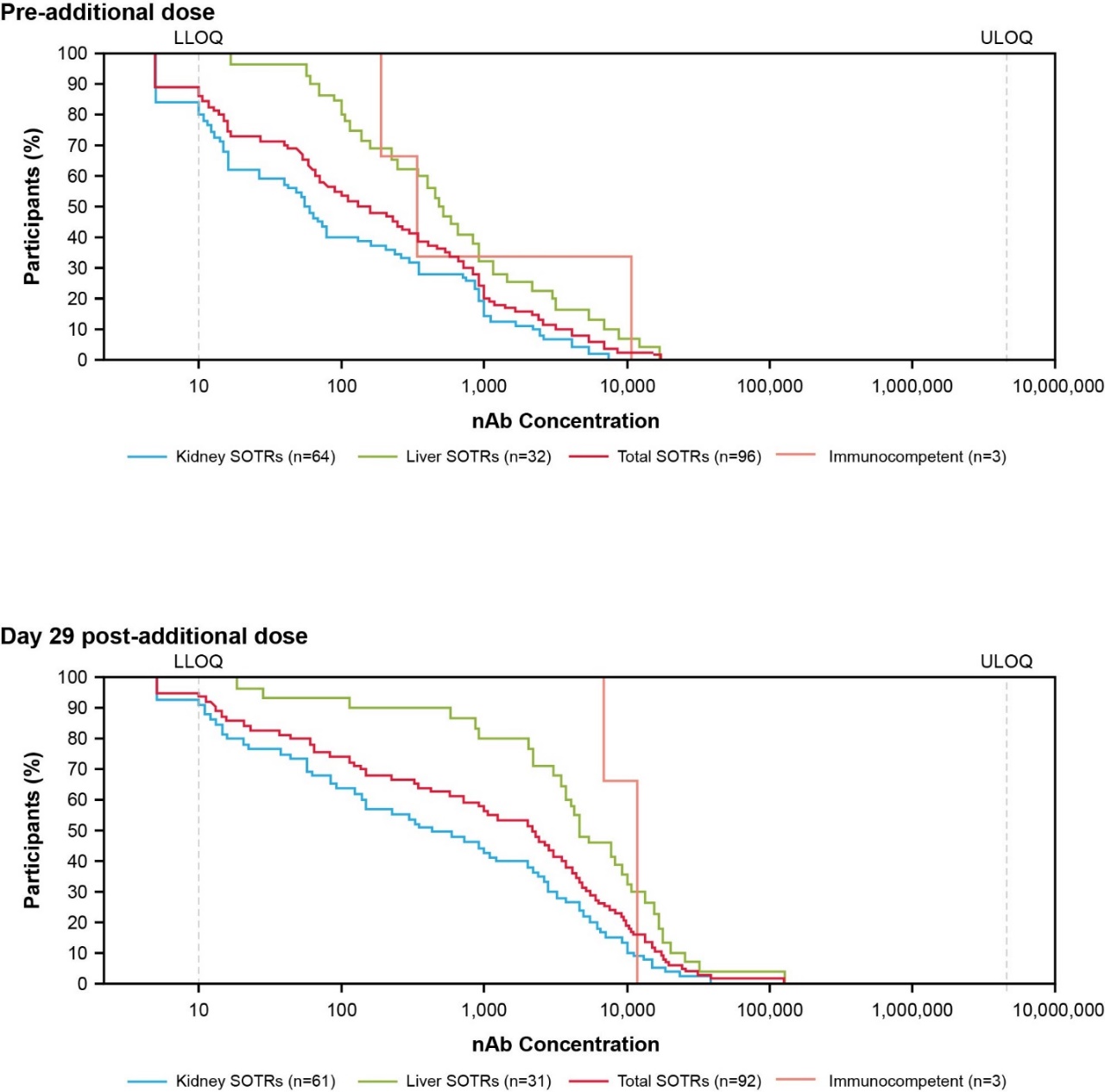

Supplement: jiae140_Supplementary_Data [file jiae140_supplementary_data.zip › Figueroa_Supplementary_Figure_S3_JID.docx]
