# Supplementary material for: Safety and Immunogenicity of the mRNA-1273 Coronavirus Disease 2019 Vaccine in Solid Organ Transplant Recipients
Source: J Infect Dis. 2024 Mar 21;230(3):e591–600. doi: 10.1093/infdis/jiae140 (PMC11420796; doi:10.1093/infdis/jiae140)
Supplement: jiae140_Supplementary_Data [file jiae140_supplementary_data.zip › Figueroa_Supplementary_Methods_Revised.docx]

# SUPPLEMENTARY METHODS

## *Participants*

For Part A, participants were eligible for inclusion if they were aged ≥18 years; had received a single kidney or liver transplant and chronic immunosuppressive therapies (ISTs) for the prevention of allograft rejection ≥90 days prior to enrollment; and were unvaccinated or previously vaccinated with 2 doses of mRNA-1273 after receipt of transplant. Immunocompetent adults were also eligible if they were ≥18 years old; in good general health; without current or previous diagnosis of an immunocompromising condition, immune-mediated disease, or other immunosuppressive condition; and had not been vaccinated with any COVID-19 vaccine.

For Part B, eligible participants were solid organ transplant recipients (SOTRs) and immunocompetent participants who were actively enrolled in Part A and ≥4 months had elapsed since the receipt of the last vaccine dose. Immunocompetent adults were also eligible if they were ≥18 years old; had received a single kidney or liver transplant and chronic ISTs ≥90 days prior to enrolment; and had completed a primary COVID-19 vaccination series (3 doses of an mRNA vaccine; 2 doses of a non–mRNA vaccine; or ≥1 dose of non–mRNA vaccine combined with 1 dose of an mRNA vaccine) after receipt of transplant. Key exclusion criteria for both parts included pregnancy or breastfeeding; acute illness or fever (body temperature ≥38.0°C/≥100.4°F); or a history of IST-associated complications.

## *Assessments*

SARS-CoV-2 infection was monitored by and clinically assessed by the investigator through the collection of nasopharyngeal swabs or other specimens at the pre-specified study visits (**Fig. S1**) and if the participant experienced any of the following: symptoms of infection; exposure to another individual with confirmed infection; or medically attended adverse event suggestive of SARS-CoV-2 infection. Specimens collected for SARS-CoV-2 testing were evaluated by serologic assay (Elecsys anti–SARS-CoV-2 binding antibody [bAb]) and by reverse transcription-polymerase chain reaction (RT-PCR). Laboratory assessment of kidney and liver function were performed monitored through 28 days after each dose to assess factors indicative of organ rejection. Events of biopsy-proven organ rejection were monitored and reported through the end of the study.

Symptomatic COVID-19 cases were defined as participants with ≥1 positive SARS-CoV-2 result on PCR testing and presentation of ≥2 of the following symptoms: fever (≥38°C/≥100.4°F), chills, myalgia, headache, sore throat, or new olfactory and taste disorder(s); or ≥1 of the following respiratory symptoms: cough, shortness of breath, difficulty breathing, or clinical or radiological evidence of pneumonia. Severe cases were defined as participants who met the definition for COVID-19 as described above and exhibited ≥1 of the following symptoms: clinical signs indicative of severe systemic illness; a respiratory rate ≥30 breaths/min; heart rate ≥125 beats/min; oxygen saturation level ≤93% or partial pressure of oxygen/fraction of inspired oxygen <300 mm Hg; respiratory failure or evidence of shock; significant acute renal, hepatic, or neurologic dysfunction; admission to an intensive care unit; or death.

Neutralizing antibody (nAb) geometric mean concentrations (GMCs) against the spike protein of ancestral (Wuhan-Hu-1) SARS-CoV-2 were quantified by a validated pseudovirus neutralization assay (PsVNA) [37]. Serum bAb geometric mean (GM) levels against ancestral SARS-CoV-2 and variant (alpha [B.1.1.7], beta [B.1351], delta [AY.4], gamma [P.1], and omicron [B.1.1.529]) spike proteins were measured by ligand-binding assay (MSD-ECL Multiplex).

## *Statistical analysis*

A sample size of 240 participants (220 unvaccinated or previously vaccinated SOTRs and 20 unvaccinated immunocompetent participants) who received the 2-dose (immunocompetent participants) or 3-dose (SOTRs) regimens or the additional dose were expected to provide estimates of antibody responses. With 200 SOTRs, if the true adverse event rate of biopsy-proven organ rejection was 3%, the study had approximately >95% probability to observe ≥1 participant reporting such an event. Descriptive statistics were used to summarize safety, immunogenicity, and effectiveness data.

Safety was assessed in the safety set, which included participants who received mRNA-1273 in Part A or Part B. Safety outcomes were also evaluated in the solicited safety set, which comprised participants contributing any solicited adverse reactions (AR) after any mRNA-1273 dose. Immunogenicity was assessed in the respective Part A and Part B per-protocol immunogenicity sets (PPISs), which comprised participants in the safety set without SARS-CoV-2 infection pre-vaccination (Part A) and before the additional dose (Part B), received planned doses per schedule, and had no major protocol deviations. Of note, after the determination of the PPIS for each study part, concomitant medications and/or vaccines received during the study period that were anticipated to impact immunogenicity evaluations were reviewed to determine the eligibility of SOTR participant samples. Immunogenicity data collected at or after the receipt of these medications and/or vaccines were subsequently excluded from the analyses. COVID-19 and severe COVID-19 incidences were assessed in the modified intent-to-treat (mITT) sets, which comprised all participants in the safety set without SARS-CoV-2 infection pre-vaccination (Part A) and before the additional dose (Part B).

For primary and secondary immunogenicity analyses, nAb GMCs, bAb GMs, and geometric mean fold rises (GMFRs) were reported with corresponding 95% confidence intervals (CIs). GMFRs were calculated as the post-baseline timepoint over pre-vaccination in Part A or pre-additional dose in Part B. CIs were calculated based on the *t* distribution of the log-transformed values, or the difference in the log-transformed values for GMC and GMFR, respectively, then back-transformed to the original scale. Seroresponse at the participant level was defined as a change in antibody levels to ≥4 × the lower limit of quantification (LLOQ) in participants with baseline levels <LLOQ and as ≥4-fold rise in antibody levels in participants with baseline levels <LLOQ. Additionally, the proportion of participants with a ≥4-fold rise of serum SARS-CoV-2–specific antibody levels from baseline at each post-baseline timepoint were summarized with 2-sided 95% Clopper-Pearson CIs.
