# Supplementary material for: Safety and Immunogenicity of the mRNA-1273 Coronavirus Disease 2019 Vaccine in Solid Organ Transplant Recipients
Source: J Infect Dis. 2024 Mar 21;230(3):e591–600. doi: 10.1093/infdis/jiae140 (PMC11420796; doi:10.1093/infdis/jiae140)
Supplement: jiae140_Supplementary_Data [file jiae140_supplementary_data.zip › Figueroa_Supplementary_Table_S1_JID.docx]

**Table S1. Summary of Unsolicited Adverse Events Reported up to 28 Days After Any mRNA-1273 Dose Among SOTRs (Safety Set)**

|  | **Kidney SOTRs n=137** | **Liver SOTRs n=77** | **Total SOTRs N=214** |
| --- | --- | --- | --- |
| **All unsolicited AEs, n (%)** |  |  |  |
| All | 49 (35.8) | 41 (53.2) | 90 (42.1) |
| Serious | 10 (7.3) | 3 (3.9) | 13 (6.1) |
| Fatal | 0 | 0 | 0 |
| Biopsy-proven organ rejection | 0 | 1 (1.3) | 1 (0.5) |
| Medically attended AEs | 35 (25.5) | 22 (28.6) | 57 (26.6) |
| AESIs | 2 (1.5) | 4 (5.2) | 6 (2.8) |
| Leading to discontinuation from participation | 0 | 0 | 0 |
| Severe | 10 (7.3) | 7 (9.1) | 17 (7.9) |
| **Unsolicited AEs related to mRNA-1273, n (%)** |  |  |  |
| All | 24 (17.5) | 22 (28.6) | 46 (21.5) |
| Serious | 2 (1.5) | 0 | 2 (0.9) |
| Fatal | 0 | 0 | 0 |
| Medically attended AEs | 5 (3.6) | 2 (2.6) | 7 (3.3) |
| AESI | 0 | 1 (1.3) | 1 (0.5) |
| Leading to discontinuation from participation | 0 | 0 | 0 |
| Severe | 6 (4.4) | 5 (6.5) | 11 (5.1) |

AE, adverse event; AESI, adverse event of special interest; SOTR, solid organ transplant recipient.
