# Supplementary material for: Safety and Immunogenicity of the mRNA-1273 Coronavirus Disease 2019 Vaccine in Solid Organ Transplant Recipients
Source: J Infect Dis. 2024 Mar 21;230(3):e591–600. doi: 10.1093/infdis/jiae140 (PMC11420796; doi:10.1093/infdis/jiae140)
Supplement: jiae140_Supplementary_Data [file jiae140_supplementary_data.zip › Figueroa_Supplementary_Table_S2_JID.docx]

**Table S2. Subgroup Analysis of nAb Concentrations Against Ancestral SARS-CoV-2 by Antimetabolite IST use among SOTRs in Part A and B (Per-Protocol Immunogenicity Sets)**

|  | **Antimetabolite n=36** | **No antimetabolite n=12** |
| --- | --- | --- |
| **Part A** | | |
| **Baseline (pre-additional dose)** |  |  |
| n^a^ | 32 | 8 |
| GMC (95% CI)^b^ | 15.1 (11.2, 20.3) | 11.1 (5.5, 22.4) |
| **Day 57 (post-dose 2)** |  |  |
| n^a^ | 30 | 8 |
| GMC (95% CI)^b^ | 68.9 (35.0-135.8) | 228.9 (42.2-1241.3) |
| GMFR (95% CI)^b^ | 4.4 (2.2-8.6) | 20.6 (3.2-134.0) |
| **Day 113 (post-dose 3)** |  |  |
| n^a^ | 29 | 6 |
| GMC (95% CI)^b^ | 326.2 (148.3-717.6) | 1597.9 (116.7-21881.1) |
| GMFR (95% CI)^b^ | 20.8 (9.4-45.8) | 110.4 (5.5-2218.4) |
| **Part B** | | |
| **Baseline (pre-additional dose)** |  |  |
| n^a^ | 74 | 22 |
| GMC (95% CI)^b^ | 108.7 (66.0-178.9) | 586.8 (227.0-1517.3) |
| **Day 29 (28 days post-additional dose)** |  |  |
| n^a^ | 71 | 21 |
| GMC (95% CI)^b^ | 569.1 (309.6-1046.1) | 3387.5 (1042.5-11007.7) |
| GMFR (95% CI)^b^ | 4.8 (3.4-6.8) | 5.7 (2.9-11.1) |

^a^Number of participants with non-missing data at the corresponding timepoint.

^b^95% CIs were calculated based on the *t* distribution of the log-transformed values or the difference in the log-transformed values for GMC value and GMFR, respectively, then back-transformed to the original scale for presentation.

Antibody values <LLOQ were replaced by 0.5 x LLOQ. Values >ULOQ were replaced by the ULOQ.

nAb, neutralizing antibody; CI, confidence interval; GMC, geometric mean concentration; GMFR, geometric mean fold rise (post-dose/baseline titers); LLOQ, lower limit of quantification; SOTR, solid organ transplant recipient; ULOQ, upper limit of quantification.
