# Supplementary material for: Safety and Immunogenicity of the mRNA-1273 Coronavirus Disease 2019 Vaccine in Solid Organ Transplant Recipients
Source: J Infect Dis. 2024 Mar 21;230(3):e591–600. doi: 10.1093/infdis/jiae140 (PMC11420796; doi:10.1093/infdis/jiae140)
Supplement: jiae140_Supplementary_Data [file jiae140_supplementary_data.zip › Figueroa_Supplementary_Table_S3_JID.docx]

**Table S3. Subgroup Analysis of nAb Concentrations Against Ancestral SARS-CoV-2 by Primary COVID-19 Vaccine Series Type After an Additional Dose of mRNA-1273 Among SOTRs**

|  | **3-dose mRNA-1273**  **n=74**^a^ | **3-dose BNT162b2**  **n=29** | **Other**^b^  **n=5** | **Total**  **N=108** |
| --- | --- | --- | --- | --- |
| **Baseline (pre-additional dose)** | | | | |
| n^c^ | 65 | 26 | 5 | 96 |
| GMC (95% CI)^d^ | 256.2 (147.7-444.5) | 56.4 (24.2-131.4) | 79.3 (10.2-619.5) | 160.0 (101.3-252.5) |
| **Day 29 (28 days post-additional dose)** | | | | |
| n^c^ | 64 | 24 | 4 | 92 |
| GMC (95% CI)^d^ | 1470.4 (777.6-2780.3) | 201.4 (70.6-574.3) | 857.7 (6.5-113958.8) | 855.1 (491.9-1486.5) |
| GMFR (95% CI)^d^ | 5.5 (3.9-7.7) | 3.1 (1.7-5.7) | 16.3 (0.2-1441.4) | 5.0 (3.7-6.7) |
| SRR |  |  |  |  |
| N1^e^ | 64 | 24 | 4 | 92 |
| SRR (%)^f^ | 39 (60.9) | 7 (29.2) | 3 (75.0) | 49 (53.3) |
| 95% CI^g^ | 47.9-72.9 | 12.6-51.1 | 19.4-99.4 | 42.6-63.7 |

^a^Number of participants who completed the 3-dose mRNA-1273 primary series vaccination within or outside of the study.

^b^Primary vaccination with 2 doses of a non–mRNA vaccine or ≥1 non–mRNA vaccine dose combined with an mRNA dose.

^c^Number of participants with non-missing data at the corresponding timepoint.

^d^95% CIs were calculated based on the *t* distribution of the log-transformed values or the difference in the log-transformed values for GMC value and GMFR, respectively, then back-transformed to the original scale for presentation.

^e^Number of participants with baseline values meeting the criterion at the corresponding timepoint. Percentages were based on N.

^f^Seroresponse at a participant level was defined as a change from <LLOQ to ≥4 x LLOQ, or ≥4-fold rise if baseline value was ≥LLOQ.

^g^95% CIs were calculated using the Clopper-Pearson method.

N1, number of participants with non-missing data at baseline and the corresponding post-baseline timepoint.

Antibody values <LLOQ were replaced by 0.5 x LLOQ. Values >ULOQ were replaced by the ULOQ.

CI, confidence interval; GMC, geometric mean concentration; GMFR, geometric mean fold rise (post-dose/baseline titers); LLOQ, lower limit of quantification; nAb, neutralizing antibody; SOTR, solid organ transplant recipient; SRR, seroresponse rate; ULOQ, upper limit of quantification.
